# Outcome and prognosis of hypoxic brain damage patients undergoing neurological early rehabilitation

**DOI:** 10.1186/s13104-015-1175-z

**Published:** 2015-06-17

**Authors:** Ute E Heinz, Jens D Rollnik

**Affiliations:** Institute for Neurorehabilitation Research (InFo), Medical School Hannover (MHH), BDH-Clinic Hessisch Oldendorf, Greitstr 18-28, 31840 Hessisch Oldendorf, Germany

**Keywords:** Hypoxic brain damage, Early rehabilitation, Outcome

## Abstract

**Background:**

The prevalence of patients suffering from hypoxic brain damage is increasing. Long-term outcome data and prognostic factors for either poor or good outcome are lacking.

**Methods:**

This retrospective study included 93 patients with hypoxic brain damage undergoing neurological early rehabilitation [length of stay: 108.5 (81.9) days]. Clinical data, validated outcome scales (e.g. Barthel Index—BI, Early Rehabilitation Index—ERI, Glasgow Coma Scale—GCS, Coma Remission Scale—CRS), neuroimaging data, electroencephalography (EEG) and evoked potentials were analyzed.

**Results:**

75.3% had a poor outcome (defined as BI <50). 38 (40.9%) patients were discharged to a nursing care facility, 21 (22.6%) to subsequent rehabilitation, 17 (18.3%) returned home, 9 (9.7%) needed further acute-care hospital treatment and 8 (8.6%) died. Barthel Index on admission as well as coma length were strong predictors of outcome from hypoxic brain damage. In addition, duration of vegetative instability, prolongation of wave III in visual evoked potentials (flash VEP), theta and delta rhythm in EEG, ERI, GCS and CRS on admission were related to poor outcome. All patients with bilateral hypodensities of the basal ganglia belonged to the poor outcome group. Age had no independent influence on functional status at discharge.

**Conclusions:**

As with other studies on neurological rehabilitation, functional status on admission turned out to be a strong predictor of outcome from hypoxic brain damage.

## Background

Hypoxic brain damage, also called hypoxic–ischemic encephalopathy, is a severe consequence of global cerebral ischemia due to cardiac arrest [1] or other causes (e.g. hanging, strangulation, poisoning with carbon monoxide or near-drowning). Cardiac diseases are the main cause of cardiac arrests (82.4%) and subsequent brain damage [2]. In the United States, approximately 180.000–450.000 people (in Europe about 270.000 people) are dying because of sudden cardiac death per year [1, 2]. Due of improvement of pre-hospital emergency care, the prevalence of patients surviving resuscitation and suffering from severe hypoxic brain damage is increasing [3].

The spectrum of disability resulting from hypoxic-ischemic encephalopathy ranges from complete recovery to coma or even death [3, 4]. Clinical trials showed that 27% of post-hypoxic coma patients regained consciousness within 28 days, 9% remained comatose or in an unresponsive wakefulness syndrome (UWS), and 64% died [3, 4]. In another prospective clinical study, 18.6% of patients stayed in an UWS [5].

In intensive care medicine, in particular in neurological early rehabilitation, there is a need for reliable criteria indicating either good or poor prognosis. Several studies tried to define outcome criteria in the acute phase of postanoxic coma. Outcome criteria in hypoxic brain damage may be divided into three different categories: reliable variables to predict poor outcome, variables related to poor outcome, and variables of unclear prognostic value [6]. The usefulness of clinical, biochemical, neuroimaging and neurophysiological data as outcome predictors is discussed in the following paragraphs.

### Clinical data

In recent studies, variables like aetiology of cardiac arrest, duration of hypoxia and resuscitation circumstances are discussed controversially. However, these parameters are without sufficient predictive value. Resuscitation characteristics (e.g. duration of anoxia or resuscitation) and hyperthermia appear to be the only variables related to poor outcome [6]. In addition, it has been shown that reliable clinical predictors are absent papillary reactions to light, absent corneal reflexes and absent or extensor motor responses to pain [6].

A large study enrolling 97 patients after cardiopulmonary resuscitation showed that unconsciousness for more than 48 h and Glasgow Coma Scale (GCS) less than 6 after 72 h are predictors of a poor neurological outcome [5].

### Biochemical parameters

Serum neuron-specific enolase (NSE) was found to be a specific factor for poor outcome, with a cut-off >33 ng/ml in a big prospective multicentre study [2]. However, biochemical variables (e.g. serum neuron-specific enolase, NSE) are outlined as variables with unclear prognostic value [6]. In another study, serum NSE and S-100 protein were found to be reliable predictors in combination with GCS, only [5]. In summary, biochemical factors are discussed controversially.

### Neuroimaging

Two out of three cases with bilateral basal ganglia hypodensities, as a typical CT finding of acute global cerebral hypoperfusion, were discharged without severe neurological impairment [4]. Further CT findings are: diffuse mass effect with effacement of the cerebral sulci and of the brain stem cisterns, loss of the normal gray-white matter differentiation due to edema and a decreased density in a watershed distribution bilaterally [4]. In magnetic resonance imaging (MRI), controlled studies with large numbers of patients after cardiac arrests are lacking 111[7]. Brain swelling, cortical laminar necrosis, hyperintensity of basal ganglia, delayed white matter degeneration and atrophy may be MRI findings of hypoxia [7]. During the acute and subacute phase, diffusion weighted imaging (DWI) and T2-weighted sequences show hyperintense signals in cortex, thalamus and basal ganglia. In the subacute phase, hyperintense signals decrease and white matter abnormalities occur [7]. In the chronic phase, a diffuse atrophy and hydrocephalus may be observed [7]. Neuroimaging data investigating prognostic factors for hypoxic brain damage are still rare. Thus, neuroimaging findings in cranial CT or MRI are outlined as variables with unclear prognostic value [6].

### Clinical neurophysiology

The prognostic value of burst-suppression or isoelectric electroencephalography (EEG) is discussed controversially [6, 8]. However, a burst-suppression pattern is frequently associated with poor outcome [8]. Reliable predictors are: status epilepticus within the first 24 h and bilateral absence of cortical median nerve somatosensory evoked potentials (SSEP) during the first 3 days [6].

Long-term outcome data of patients with hypoxic brain damage are still rare. In a study with 46 comatose patients (observation period 2 years), responsive patients were significantly younger and had lower Disability Rating Scale and higher Coma Recovery Scale (CRS) scores at study entry [9].

In Germany, many patients suffering from hypoxic–ischemic encephalopathy enter neurological early rehabilitation, an inpatient treatment for patients with severe brain damage [10]. A study with 2060 early rehabilitation patients demonstrated, that cases with hypoxic–ischemic encephalopathy had a notably longer mean length of stay (LOS: 56 versus 44.6 days) [10].

Only one study is available on rehabilitation outcome of hypoxic-ischemic patients [11]. The study enrolled 113 inpatient cases and mainly focused on recovery of consciousness, but not on functional abilities. Only 19.5% of patients regained consciousness during inpatient rehabilitation [11]. However, there is a considerable lack of evidence regarding rehabilitation outcome and prognostic factors. In particular, it is unclear which patients benefit from neurological early rehabilitation. This lack of evidence motivated the authors of the present study to review cases of hypoxic brain damage patients and to define outcome predictors.

## Methods

### Patients

Medical records of 93 neurological rehabilitation cases (68 male, 25 female) with hypoxic–ischemic encephalopathy were reviewed. The patients were admitted to the BDH Clinic Hessisch Oldendorf, Germany, a large neurological early rehabilitation facility, from 2004 to 2008. Criterion for admission to early rehabilitation was completion of acute hospital care (i.e. cardiorespiratory and intracranial pressure stability). Neurological early rehabilitation offers a standardized treatment, including 300 min of therapeutic interventions per day (e.g. physiotherapy, occupational therapy, speech/swallowing therapy and specialized nursing care) [10]. Patients were discharged if there was a failure to make further improvement or if complications occurred which could only be treated in an acute hospital setting (e.g. gastrointestinal bleeding). In addition, death was an endpoint. Mean age of hypoxic patients was 53.0 (14.6) years (range 15–77), mean length of stay (LOS) 108.5 (81.9) days (range 1–535). Causes of hypoxic–ischemic encephalopathy were mainly cardiac diseases (30.1% myocardial infarction, 4.3% cardiac arrhythmia) or unknown (24.7%), Table 1.

**Table 1:** Cause of hypoxic brain damage

Etiology	n	%
Cardiac infarction	28	30.1
Cardiac arrhythmia	4	4.3
Pulmonary embolism	4	4.3
Respiratory insufficiency	3	3.2
Attempted suicide	3	3.2
Qt syndrome	2	2.2
Heart injuries	2	2.2
Intoxication	2	2.2
Anaphylactic shock	1	1.1
Status epilepticus	1	1.1
Unknown	23	24,7
Other cause	20	21.5
Sum	*93*	*100*

### Measures/procedures

As clinical data, we analyzed morbidity (co-diagnoses), duration of cardiac arrest (hypoxic interval), length and type of resuscitation (professional versus non-professional). In addition, the time until the first signs of recovery were detected (coma length) and duration of vegetative instability (e.g. hyper/hypotension, perspiration) was analyzed. Disorders of consciousness were defined as: no impairment (completely aware), confusion, somnolence, sopor (reacts to painful stimuli), and coma (unresponsiveness) [12].

### Endocrinological parameters

Serum levels of prolactin, testosterone, somatomedin, cortisol, thyroid-stimulating hormone (TSH), thyroid hormones (T3, T4) were analyzed in a few cases, only. TSH serum values, for instance, were examined in 35 cases (37.6%).

### Neuroimaging

**Figure 1:**
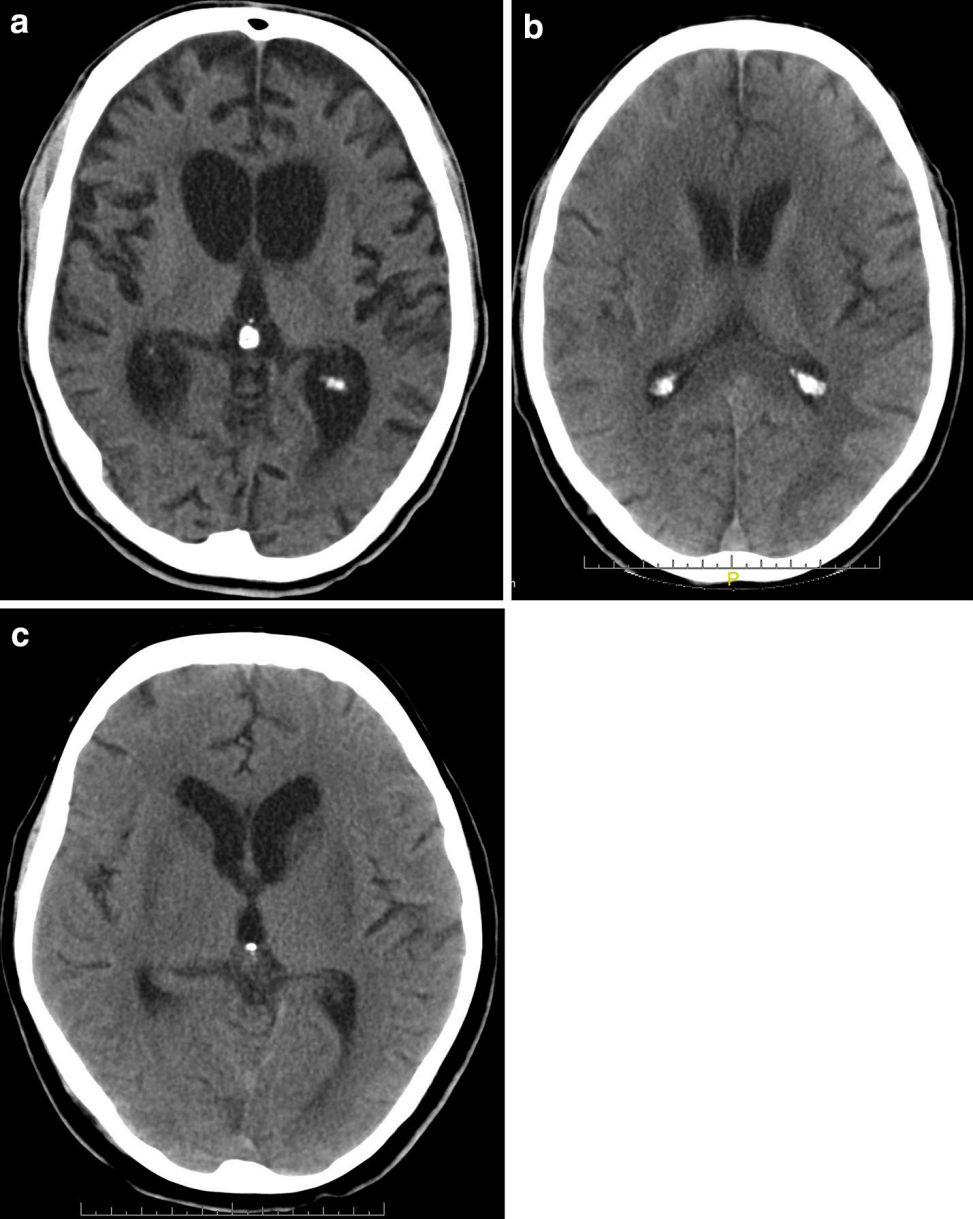
Typical CT scans of hypoxic brain damage patients. **a** Brain atrophy and hydrocephalus of a 44 y old male patient, 6 months after hypoxia. The patient was in a minimally conscious state (MCS). **b** Hypodense white matter changes of a 63 y old male patient, 2 weeks after hypoxic brain damage. The patient was in an unresponsive wakefulness syndrome (UWS). **c** Bilateral basal ganglia hypodensities of a 61 y old female patient, 6 weeks after hypoxic brain damage. The patient was in a MCS.

CT-scans were evaluated for hydrocephalus, cerebral edema, hypodensities in the cortex and in the basal ganglia. Typical CT findings are shown in Figure 1a–c. In seven cases, MRI scans were available and findings were included in the analysis.

### Clinical neurophysiology

**Figure 2:**
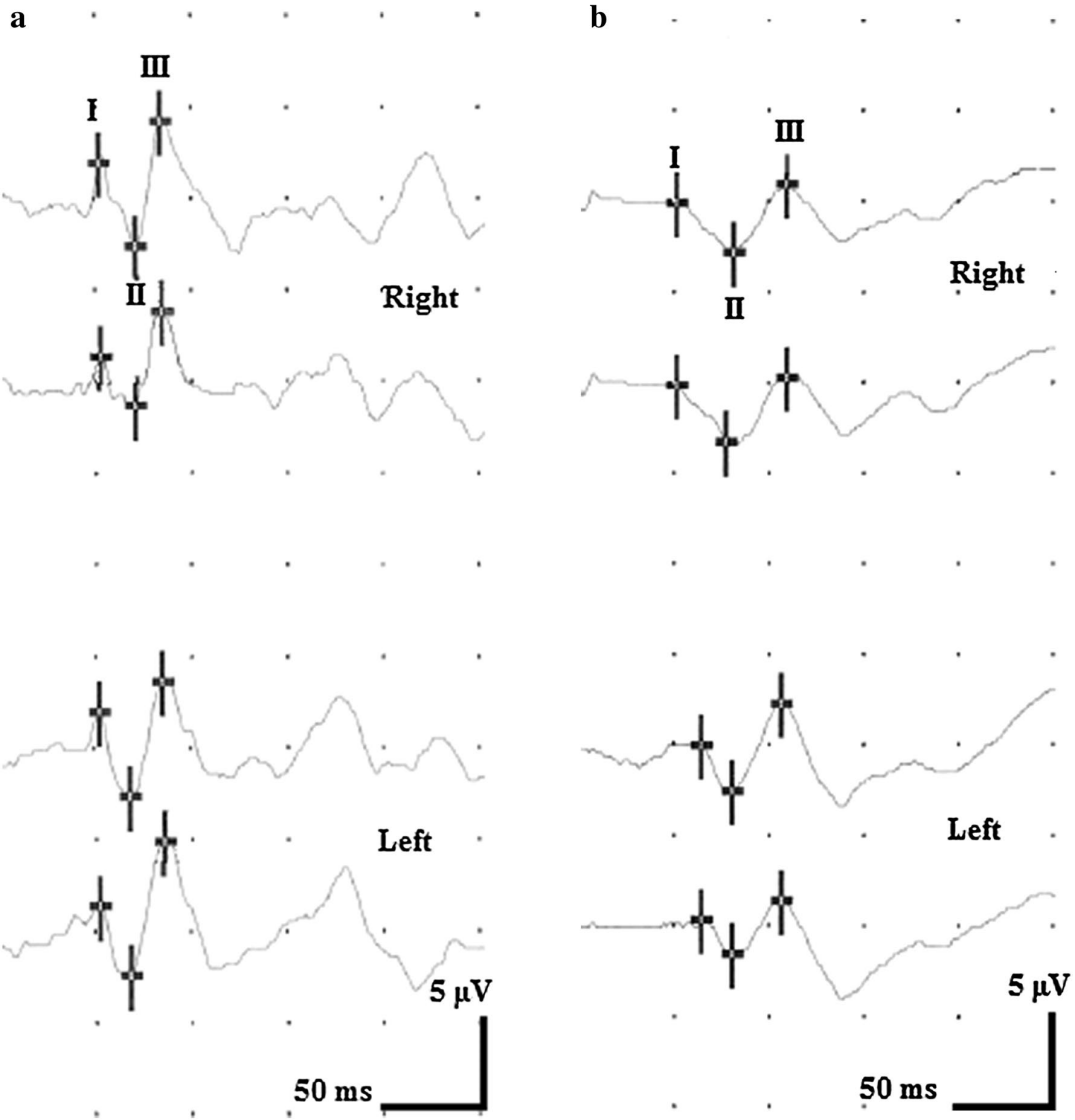
Flash VEP of hypoxic brain damage patients. **a** Flash VEP of a 60 y old male with good outcome. Latency III is 85 ms on the right and 86 ms on the left side. **b** Flash VEP of a 50 y old male with poor outcome. Compared to the example in **a**, latencies I and II are not different, but latency III is delayed on both sides (108 ms right, 107 ms left).

Electroencephalography (EEG), auditory evoked potentials (AEP), visual evoked potentials (VEP), somatosensory evoked potentials (SSEP) of the median nerve were recorded usually within the first 2 weeks after admission. EEG was done using the international 10/20 system (Neurofax EEG 9000, Nihon Kohden Europe, Rosbach, Germany). Surface electrodes were used for evoked potentials (Nicolet Viking Select, Natus Medical, Middleton, WI, USA). VEPs were done with flashing light-emitting diodes (flash VEP, stimulation frequency 1.3 Hz). Latencies and amplitudes of waves I–III were examined according to the guidelines of the American Clinical Neurophysiology Society [13], Figure 2a, b. In addition, AEP latencies I–V and N20/P25 latencies and amplitudes of median nerve SSEPs were analyzed.

### Validated outcome scales

Barthel Index (BI) [14] and Early Rehabilitation Index (ERI) [15] were used to measure functional ability. BI ranges from 0 (= completely dependent on nursing) to 100 (= functional independence). ERI consists of items like mechanical ventilation, tracheostomy, or dysphagia and ranges from −325 (minimum) to 0 (maximum). Glasgow Coma Scale (GCS) [16] and Coma Remission Scale (CRS) [17] were used to measure depth of coma. GCS minimum is 3 (deep coma) and maximum score 15 points (completely aware). CRS ranges from 0 (minimum) to 24 (maximum) allowing a more detailed evaluation of coma depth. In line with previous studies, poor outcome was defined as BI <50 [18].

### Statistics

For statistical analyses, SPSS™ 21.0 software package (SPSS Inc, Chicago, USA) was used. In the results section, mean values and standard deviations (in brackets) are displayed. T tests for independent samples were used to compare poor and bad outcome groups. A univariate ANOVA model was designed, with BI at discharge as dependent variable, and age, BI on admission, GCS on admission, coma length, ERI and CRS on admission as independent covariates. For correlational analyses, bivariate Pearson correlations were conducted. In addition, χ^2^-tests were used for categorical predictors. Differences were regarded as significant with *p* < 0.05.

## Results

### Clinical data

**Figure 3:**
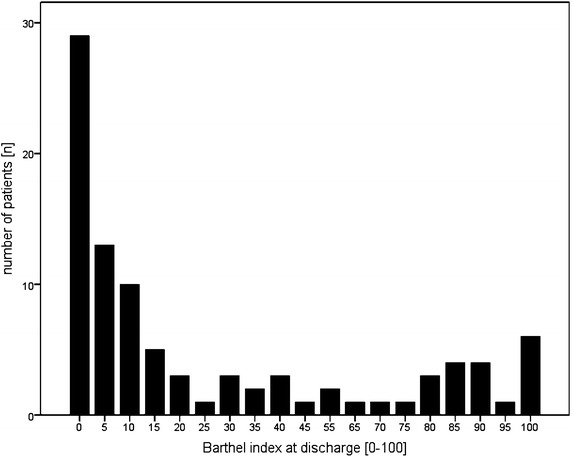
Histogram Barthel-Index at discharge.

**Figure 4:**
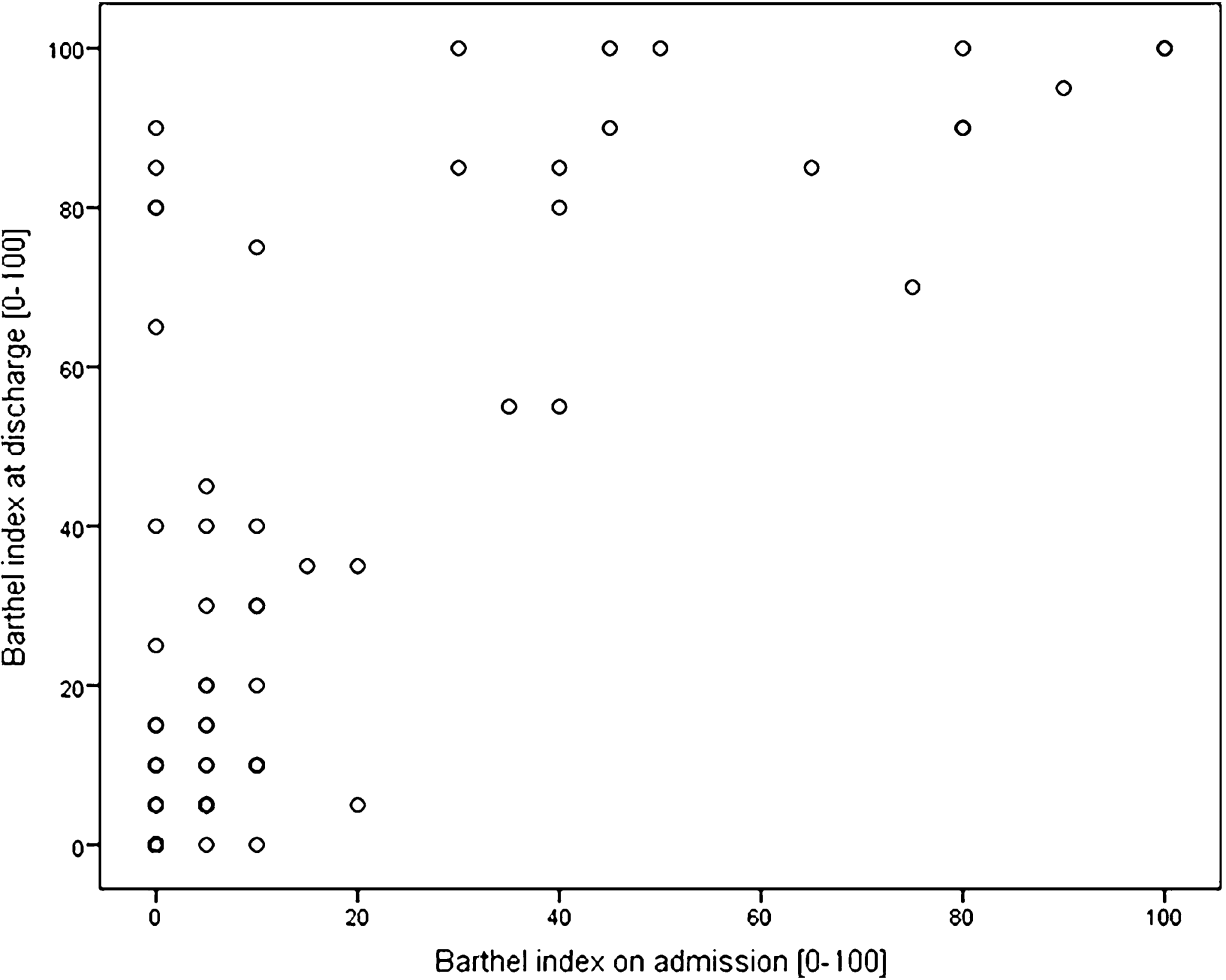
Scatter plot showing Barthel Index (BI) on admission and at discharge (r = 0.759, *p* < 0.001).

Among 93 cases, 70 early rehabilitation patients (75.3%) had a poor (BI <50) and only 23 (24.7%) a good outcome. Histogram of BI at discharge is displayed in Figure 3. Figure 4 shows a scatter plot of BI on admission and at discharge. Low BI on admission (poor functional status) was correlated with low BI at discharge (r = 0.759, *p* < 0.001). Discharge placement: 38 (40.9%) patients were discharged to a nursing care facility, 21 (22.6%) to subsequent rehabilitation, 17 (18.3%) returned home. Nine (9.7%) needed acute-care hospital treatment (e.g. neurosurgical intervention) and 8 (8.6%) died. Disorders of consciousness were observed in 77 cases (82.8% of all cases) on admission, Table 2. Cases with no impairment of consciousness increased from 16 (17.2%) on admission to 34 (36.6%) at discharge. However, 23 out of 28 patients (82.1%) who were comatose on admission stayed comatose at discharge.

**Table 2:** Disorders of consciousness on admission and at discharge

		DOC at discharge					Sum
		No impairment	Confusion	Somnolent	Sopor	Coma	
DOC on admission	No impairment	16	0	0	0	0	*16*
	Confusion	13	20	0	0	0	*33*
	Somnolent	3	4	7	0	0	*14*
	Sopor	1	0	0	1	0	*2*
	Coma	1	1	2	1	23	*28*
Sum		*34*	*25*	*9*	2	*23*	*93*

The following parameters had no significant influence on the patients’ outcome: age, type (professional vs. non-professional) and duration of resuscitation, hypoxic interval and time until onset of neurological early rehabilitation. The entire length of neurological impatient rehabilitation did not differ significantly, either, Table 3. However, the length of stay (LOS) in early rehabilitation was considerably longer (twice) among patients with a poor outcome compared to those with good outcome, Table 3. A predictor of poor outcome was coma length. In addition, the duration of vegetative instability was significantly longer among poor outcome subjects, Table 3.

**Table 3:** Characteristics of neurological early rehabilitation patients with good and poor outcome

	Good outcome (BI ≥ 50)	Poor outcome (BI < 50)	*p*-value*
N	23	70	n.a.
Age [years]	50.9 (12.5)	53.7 (15.2)	n.s.
Time interval until onset of neurological early rehabilitation [days]	26.0 (17.1)	34.0 (39.2)	n.s.
LOS—neurological early rehabilitation [days]	46.4 (68.2)	95.4 (53.2)	<0.01
LOS—inpatient neurological rehabilitation [days]	98.8 (86.9)	111.7 (80.6)	n.s.
Number of co-diagnoses [n]	10.4 (5.0)	17.3 (6.0)	<0.001
Hypoxic interval [min]	8.8 (6.3)	7.4 (6.0)	n.s.
Duration of resuscitation [min]	30.0 (–)	23.5 (10.0)	–
Time until signs of recovery observed (coma length) [days]	17.6 (18.9)	46.8 (44.6)	<0.01
Duration of vegetative instability [days]	3.1 (6.7)	24.2 (36.6)	<0.01
Barthel Index (BI) on admission [0 to 100]	45.0 (33.7)	3.3 (4.8)	<0.001
BI at discharge [0 to 100]	85.0 (13.7)	9.3 (12.2)	<0.001
Delta BI (discharge minus admission)	40.0 (30.6)	6.0 (10.7)	<0.001
Early Rehabilitation Index (ERI) on admission [−325 to 0]	−112.0 (79.4)	−188.6 (95.0)	<0.01
ERI at discharge [−325 to 0]	−38.0 (49.4)	−144.3 (87.1)	<0.001
Delta ERI (discharge minus admission)	73.9 (17.7)	44.3 (8.7)	n.s.
Delta ERBI (Early Rehabilitation Barthel Index, discharge minus admission)	113.9 (109.7)	50.3 (75.4)	<0.01
Coma Remission Scale (CRS) [0 to 24] on admission	20.8 (5.6)	11.8 (8.3)	<0.001
Glasgow Coma Scale (GCS) on admission [3 to 15]	12.2 (2.1)	8.6 (3.2)	<0.001
Glasgow Coma Scale (GCS) at discharge [3 to 15]	13.7 (0.6)	9.1 (3.6)	<0.001

* t-tests for independent samples, *n.a.* not applicable, *n.s.* not significant (*p* > 0.05).

Morbidity (number of co-diagnosis), BI on admission and at discharge, ERI at discharge, CRS, and GCS showed highly significant differences between groups, Table 3.

Serum-levels of endocrinological parameters did not differ between good and poor outcome groups, Table 4.

**Table 4:** Endocrinological parameters

Hormones	Good outcome	Poor outcome
TSH [mU/ml)	1.53 (0.68)	1.37 (1.18)
T3 [ng/ml]	1.00 (0.20)	0.91 (0.27)
T4 [ng/dl]	1.10 (0.34)	1.35 (0.35)
Prolactin [µg/l]	20.00 (–)	13.52 (9.08)
Testosterone [µg/l]	3.70 (–)	2.23 (1.58)
Oestradiol [ng/l]	– (–)	16.00 (2.83)
Cortisol [µg/l]	194.00 (–)	193.00 (68.69)
Somatomedin C [µg/l]	179.00 (–)	197.81 (122.22)

There were no significant differences between good and poor outcome groups.

The following correlations were observed: the hypoxic interval correlated positively with a higher level of TSH indicating hypothyroidism (r = 0.58, *p* < 0.05). The longer the resuscitation time, the longer the LOS in the primary (referring) hospital (r = 0.54, *p* < 0.05). Further, there was a positive correlation between the time interval until remission signs were observed and LOS in the referring hospital (r = 0.46, *p* < 0.05) and in rehabilitation (r = 0.33, *p* < 0.05).

Changes in BI (discharge minus admission) correlated positively with the following factors: GCS on admission (r = 0.32, *p* < 0.01) and at discharge (r = 0.50; *p* < 0.01), coma length (r = 0.25, *p* < 0.05) and CRS on admission (r = 0.34, *p* < 0.01). GCS on admission (*p* < 0.01; r = 0.88) and at discharge (*p* < 0.01; r = 0.74) correlated positively with CRS. In addition, there was a positive correlation between GCS on admission and ERI on admission (r = 0.52, *p* < 0.01) and at discharge (r = 0.46, *p* < 0.01). The higher the CRS the higher was the ERI on admission (r = 0.59, *p* < 0.01) and at discharge (r = 0.52, *p* < 0.01).

Age correlated negatively with LOS in rehabilitation (r = −0.26, *p* < 0.05), and positively with CRS (r = 0.21, *p* < 0.05).

A univariate ANOVA model was designed with BI at discharge as dependent variable, age, BI on admission, GCS on admission, coma length, ERI on admission, and CRS on admission as independent covariates (F = 23.2, *p* < 0.001). The model explained 67.9% of data variation. Only BI on admission (*p* < 0.001) and coma length (*p* < 0.05) had a significant influence on BI at discharge. Age and duration of vegetative instability tended to have an impact but did not reach a level of significance (*p* < 0.10).

### Imaging results

In 62 cases neuroimaging was available. A hydrocephalus was detected in 37 (59.7% of all patients with neuroimaging), cerebral edema in 6 (9.7%), bilateral hypodensities of the basal ganglia in 17 (27.4%) and hypodensities of the cortex in 38 cases (61.3%). All patients with bilateral hypodensities of the basal ganglia belonged to the poor outcome group (17/38 poor, 0/7 good outcome, χ^2^ = 2.98, *p* < 0.10). In addition, patients with basal ganglia impairment showed a significantly lower ERI at discharge: −201.5 (102.9) vs. −121.1 (81.7), *p* < 0.01.

### Clinical neurophysiology

Alpha rhythm was found in 16 (17.2%), beta in 6 (6.5%), theta in 33 (35.5%), and delta in 9 (9.7%) cases. Patients with a good outcome had alpha more often (10/16) and less frequently theta (10/33) or delta rhythms (0/9) than subjects with a poor outcome; χ^2^ = 10.6 (*p* < 0.01).

Data of evoked potentials are displayed in Table 5. The only significant differences between good and poor outcome group could be found in wave III latency of flash VEP and peak-to-peak amplitude of N20/P25 (stimulating the left side). Subjects with a poor outcome hat significantly longer III latencies in flash VEP on both sides (Figure 2a, b) and lower N20/P25 amplitudes in median nerve SSEP. VEP latency III did not correlate significantly with GCS, CRS or EFA.

**Table 5:** Data of evoked potentials

Outcome	Good		Poor	
	Left	Right	Left	Right
Auditory evoked potentials (AEP)				
Latency I [ms]	1.7 (0.2)	1.7 (0.2)	1.8 (0.2)	1.8 (0.2)
Latency II [ms]	3.0 (0.1)	2.9 (0.2)	2.9 (0.3)	3.0 (0.2)
Latency III [ms]	4.0 (0.1)	4.0 (0.1)	4.0 (0.3)	4.1 (0.2)
Latency IV [ms]	5.2 (0.2)	5.1 (0.2)	5.2 (0.3)	5.2 (0.4)
Latency V [ms]	6.0 (0.2)	5.9 (0.2)	6.0 (0.3)	6.0 (0.4)
Visual evoked potentials (flash VEP)				
Latency I [ms]	56.6 (13.1)	55.0 (12.7)	54.4 (13.5)	54.3 (13.7)
Latency II [ms]	82.1 (17.9)	77.8 (12.4)	83.1 (16.2)	81.1 (14.9)
Latency III [ms]	102.7 (14.2)*	103.0 (11.3)**	125.3 (25.7)*	125.2 (25.1)**
Amplitude I/II [µV]	7.2 (8.7)	6.1 (4.7)	5.9 (5.9)	5.6 (5.8)
Amplitude II/III [µV]	7.6 (6.0)	7.1 (4.9)	9.0 (9.2)	10.0 (11.7)
Somatosensory evoked potentials (SSEP) of the median nerve				
N20 [ms]	19.6 (1.0)	20.2 (1.5)	20.7 (2.0)	21.3 (2.7)
P25 [ms]	23.4 (2.0)	24.1 (2.2)	23.9 (2.2)	24.5 (2.6)
Amplitude N20/P25 [ms]	3.5 (2.1)**	6.1 (4.7)	1.7 (1.4)**	5.6 (5.8)

Significant differences between subjects with good and poor outcome are indicated as follows: * *p* < 0.05, ** p < 0.01 (t-tests).

## Discussion

Hypoxic brain damage may be the cause of severe disability [3]. Because of improving pre-hospital and intensive care, more patients survive acute-care treatment [3] and may enter neurological early rehabilitation facilities. Compared to previous studies from acute-care hospitals, the present study had a larger sample size and a longer observation period, due to longer LOS in inpatient rehabilitation. At this point, one limitation of the present study needs to be addressed. This is a retrospective data analysis, only. Thus, LOS shows a huge variability and there was no attempt to assess outcome at a particular time-frame post hypoxic brain damage.

In the present study, poor outcome was defined as BI at discharge lower than 50 points. This definition is in line with previous studies on outcome after stroke, but may be discussed controversially [18, 19]. To the authors` experience, a BI of 50 equals a satisfactory degree of patients` functional independence. With a BI of more than 30, patients have already finished early rehabilitation and enter subsequent rehabilitation [20]. Not surprisingly, approximately three-fourths of our patients belonged to the poor outcome group at discharge.

### Clinical data

All four clinical scales used in this study (BI, ERI, CRS, GCS) showed highly significant differences between both groups on admission. We know from literature that GCS is of some prognostic value in acute-care treatment [5]. Our results suggest that GCS might also serve as a prognostic variable in neurological early rehabilitation. The other clinical scales (BI, ERI) suggest that a low functional status on admission is a strong predictor of poor outcome in hypoxic encephalopathy which is in line with previous studies on neurological early rehabilitation [15, 20, 21]. In addition, poor outcome patients had significantly more co-diagnoses suggesting that their morbidity was higher. This finding is also in line with findings from the literature [21]. The clinical scales showed significant correlations (e.g. changes in BI correlated with GCS). This finding suggests that these scales are valid instruments in the evaluation of early rehabilitation patients. Poor outcome hypoxic encephalopathy patients also had a significantly longer LOS in early rehabilitation. This finding could be explained by worse functional status and higher morbidity on admission. We know from previous studies that morbidity and functional independence are strong predictors of LOS [21, 22]. CRS may be of some value to predict outcome of inpatient rehabilitation patients [11].

In neurological rehabilitation outcome studies, age emerges as an important and independent prognostic factor (e.g. in stroke outcome) [23]. Interestingly, age was not of prognostic value in the present study. How can this finding be explained? First of all, hypoxic encephalopathy usually is a more severe and disabling disease than stroke. Thus, findings cannot be compared easily. Further, patients in the present study were younger (good outcome: 51, poor outcome: 54 years) than stroke or other neurological patients. In a previous study on neurological early rehabilitation patients, mean age was considerably higher with 62.0 years [20]. There is evidence that rehabilitation outcome worsens with increasing age [21, 23].

Hypoxic interval, which is associated with poor outcome in literature [6], had no significant influence on our patients´ outcome in our study, either. It has to be pointed out that it is very difficult to determine the hypoxic interval accurately, in particular when patients are resuscitated outside the hospital. In addition, the present study focused on early rehabilitation and not on acute-care patients with hypoxic encephalopathy. The outcome and prognostic factors relevant to acute-care hospitalized subjects might be different from ours because only survivors enter neurological early rehabilitation. This accounts for a different sample of patients.

Not surprisingly, time interval until first remission signs from coma could be observed (coma length) was significantly shorter among good outcome patients. Nevertheless, 23 out of 28 (82%) comatose patients stayed comatose during inpatient rehabilitation. This finding is in line with a previous study which demonstrated that only 19.5% regained consciousness during rehabilitation [11]. In addition, duration of vegetative instability was significantly longer in the poor outcome group. These results suggest that—besides functional ability tests such as Barthel index—coma length may be an indicator of brain damage severity and is linked to the outcomes. Patients remaining in coma can be found in the poor outcome group. They account for the little changes of functional independence from admission to discharge in this group partially.

### Endocrinological parameters

This study does not support the hypothesis that endocrinological parameters like TSH, prolactin, cortisol or somatomedin have any influence on the outcome of hypoxic brain damage patients. Only little data is available on hormonal changes in postanoxic encephalopathy [24]. It is known from brain injury and subarachnoid hemorrhage patients that somatotropic, gonadotropic and thyroid hormone disturbances occur early after trauma resp. bleeding (within the first days) [25–27]. Low thyroid hormone levels at day four may be associated with poor 3-month-outcome [27]. However, brain injury and hypoxic encephalopathy are different diseases and our patients have been examined approximately 4 weeks after the hypoxic brain damage. It may be hypothesized that the time interval to detect hormonal changes already had passed. In addition, it has to be pointed out that only few patients in our retrospective study had endocrinological tests, TSH for instance has been examined in 37.6% of all cases. The small sample size accounts for only little statistical power.

### Neuroimaging

Neuroimaging data is of limited prognostic value [4]. While most of the acute-care patients with bilateral basal ganglia hypodensities appear to have a favourable outcome [4], all early rehabilitation patients with this finding belonged to the poor outcome group. In addition, patients with basal ganglia impairment had worse ERI values at discharge. Findings from previous studies may not be used to evaluate our findings, because these data originate from acute-care hospitals and focus on the acute phase of postanoxic encephalopathy.

### Clinical neurophysiology

The finding that good outcome patients had more often alpha and less frequently theta or delta rhythms in the EEG than subjects with a poor outcome is in line with previous studies [8]. Analyzing the evoked potentials data, we found that poor outcome patients had a significantly longer III latency in flash VEP. It is well known that peaks in flash VEP may vary between individuals and are dependent on the level of arousal [13]. However, latency III did not correlate with scales of consciousness like GCS, CRS or EFA. The observed delay of wave III could indicate a more severe cortical dysfunction in patients with poor outcome.

The results from our study suggest that functional status (BI on admission) and the interval until first remission signs from coma occur are predictors of outcome from hypoxic brain damage in early rehabilitation. In addition, duration of vegetative instability, prolongation of wave III in VEP, theta and delta EEG rhythms, ERI, GCS and CRS on admission are of some prognostic value and may indicate poor outcome. This finding suggests that patients with a certain degree of independence on admission will improve even more. On the other hand, patients with low BI on admission are more likely to be dependent on help (less functional independence). Thus, one might ask whether neurological early rehabilitation makes any sense at all. It has been demonstrated that early rehabilitation improves outcome of severely impaired neurological and neurosurgical patients [20, 28]. Regardless functional independence, 18 initially comatose patients regained some degree of consciousness and 5 out of 28 patients really “awoke” from coma. In addition, we learn from our daily work that even little improvement of patients’ independency leads to palpable improvement in the patients’ quality of life. Further, it has to be taken into account that BI only has moderate change sensitivity and little improvement may not be detected by BI or ERI [15, 22].

## Conclusions

In summary, functional status on admission as well as coma length are strong predictors of outcome from hypoxic brain damage in neurological early rehabilitation. In addition, duration of vegetative instability, prolongation of wave III in VEP, theta and delta EEG rhythm, ERI, GCS and CRS on admission may indicate poor outcome. Further, all patients with bilateral hypodense lesions in the basal ganglia (CT scans) had a poor outcome. Results from this study may not be compared to findings from acute-hospital care. It has to be taken into account that only survivors of postanoxic coma enter neurological early rehabilitation accounting for a different patient sample. Currently, there is still a considerable lack of evidence. Due to the increasing incidence and severity of hypoxic brain damage, further studies on this topic are strongly encouraged.
