# Erratum to: Outcome and prognosis of hypoxic brain damage patients undergoing neurological early rehabilitation

**DOI:** 10.1186/s13104-016-2199-8

**Published:** 2016-08-09

**Authors:** Ute E. Heinz, D. Rollnik

**Affiliations:** Institute for Neurorehabilitation Research (InFo), Medical School Hannover (MHH), BDH-Clinic Hessisch Oldendorf, Greitstr 18-28, 31840 Hessisch Oldendorf, Germany

## Erratum to: BMC Res Notes (2015) 8:243 DOI 10.1186/s13104-015-1175-z

The authors would like to clarify the ethics statement in this article [1]. Before conducting this study we consulted the local ethics committee of BDH-Clinic Hessisch Oldendorf—which is not a public, official institution—and were informed that it was exempt from requiring ethics approval. However, after publication we became aware that the ethics committee of Hannover Medical School should have been consulted. Hannover Medical School ethics committee have now confirmed that they would have waived the requirement for ethics approval.
